# Distinct Roles of Muscle Strength and Postural Stability in Objective and Subjective Function in Women with Bilateral Knee Osteoarthritis

**DOI:** 10.3390/healthcare14131880

**Published:** 2026-06-27

**Authors:** Kubra Alpay, Sefa Yildirim, Elif Durgut, Ahmet Usen

**Affiliations:** 1 Department of Physiotherapy and Rehabilitation, Faculty of Health Sciences, Bezmialem Vakif University, 34050 Istanbul, Turkey; syildirim@bezmialem.edu.tr (S.Y.); edurgut@bezmialem.edu.tr (E.D.); 2 Department of Physical Medicine and Rehabilitation, Faculty of Medicine, Istanbul Medipol University, 34214 İstanbul, Turkey; ahmet.usen@medipol.edu.tr

**Keywords:** knee osteoarthritis, muscle strength, postural stability, functional performance, WOMAC

## Abstract

**Background/Objectives:** Knee osteoarthritis (OA) significantly impairs physical function and quality of life, particularly in women. Although muscle strength and postural stability are known to influence functional outcomes, their independent contributions after controlling for age, body mass index (BMI), and pain remain unclear. This study aimed to investigate the differential roles of lower extremity muscle strength and postural stability on functional status, evaluated through both performance-based tests and patient-reported outcomes, in women with bilateral knee OA. **Methods:** Sixty-four women with bilateral knee OA (mean age: 55.71 ± 5.99 years) were included in this study. Lower extremity muscle strength was assessed using the five-times sit-to-stand test, and postural stability was evaluated with the Biodex Balance System. Performance-based function was measured using the Six-Minute Walk Test (6MWT) and the stair climbing test (SCT), while self-reported function was assessed using the WOMAC function subscale (WOMAC-F). Hierarchical linear regression analyses were conducted, controlling for age, BMI, and pain. **Results:** Muscle strength emerged as the strongest independent predictor of performance-based outcomes, significantly contributing to both SCT (β = 0.330, *p* = 0.005) and 6MWT (β = −0.409, *p* = 0.001). In the 6MWT model, the effects of age and BMI became non-significant after the inclusion of muscle strength, indicating a mediating role. In contrast, self-reported function (WOMAC-F) was primarily associated with pain (β = 0.385, *p* = 0.001) and postural stability (β = 0.243, *p* = 0.040), while muscle strength showed no significant contribution. **Conclusions:** Muscle strength is the primary determinant of objective functional performance, whereas pain and postural stability are more influential in shaping perceived functional limitations. These findings highlight a dissociation between actual performance and patient-reported function. Rehabilitation strategies should prioritize strength training to improve physical performance, while also addressing pain and balance to enhance patients’ perceived function.

## 1. Introduction

Knee osteoarthritis (OA) is a degenerative joint disease characterized by progressive degeneration of articular cartilage, synovial inflammation, and structural alterations in periarticular and subchondral bone. Its multifactorial etiology encompasses mechanical stress, age-related tissue changes, and genetic predisposition [1]. Knee pain is the most prominent clinical symptom of knee OA and represents a major contributor to functional limitations in affected individuals. As the disease progresses, pain and associated physical impairments frequently interfere with activities of daily living, thereby compromising physical function and overall quality of life [2,3].

Lower-limb muscle weakness, particularly of the quadriceps, is highly prevalent in individuals with knee OA and plays a crucial role in knee joint dysfunction. The muscles surrounding the knee are essential for dynamic joint stability during weight-bearing activities. The periarticular muscles of the lower limbs are essential for controlling the knees’ multiple degrees of freedom during weight-bearing activities. Through coordinated neuromuscular activation, particularly quadriceps–hamstrings co-contraction, these muscles regulate sagittal plane motion whilst constraining excessive frontal and transverse plane movements. Furthermore, proximal hip musculature helps regulate knee joint loading by influencing pelvic stability and lower-limb alignment. Deficits in this neuromuscular control system may impair dynamic joint stability, alter joint loading patterns, and accelerate the progression of knee osteoarthritis [4,5,6]. In addition to muscle weakness, sensorimotor deficits may impact physical function in this population. Degenerative changes in joint mechanoreceptors can impair proprioception and disrupt sensorimotor control. When compounded by chronic pain and muscle weakness, these deficits compromise joint stability and postural control, often resulting in impaired static and dynamic balance. These balance deficits are associated with an increased risk of falls and further restriction of daily activities [7,8,9,10]. Clinical and objective evaluations have demonstrated that these deficits are particularly prominent during weight-bearing transitions, such as performing sit-to-stand tasks and navigating different walking cadences, leading to compensatory strategies that further stress the joint complex [11].

Although impairments in muscle strength, balance, and physical function have been extensively documented in individuals with knee OA, the relative contribution of these factors to functional outcomes remains insufficiently understood [12,13]. Previous studies have demonstrated significant associations between quadriceps weakness and disability, as well as between impaired postural control and reduced mobility; however, these impairments frequently coexist, making it difficult to determine their independent influence on functional performance [14,15]. Furthermore, functional status in knee OA is commonly assessed using either performance-based measures or patient-reported outcomes, which may capture different dimensions of functioning. Consequently, factors associated with objective physical performance may differ from those influencing patients’ perceptions of disability. While muscle weakness and postural instability are recognized as important impairments in knee OA, their relative contribution to different aspects of functional status remains unclear [16,17]. A better understanding of these relationships may help clinicians identify the most relevant rehabilitation targets and optimize individualized treatment strategies.

Therefore, the aim of this study was to investigate the differential roles of lower extremity muscle strength and postural stability on functionality, evaluated through both performance-based and self-reported outcomes, in women with bilateral knee osteoarthritis. In light of these objectives, we hypothesized that (1) lower extremity muscle strength and postural stability would be associated with objective performance-based tests compared to subjective self-reported measures, and that (2) the relationships between demographic or baseline clinical impairments and functional capacities would be indirectly mediated by specific physical components.

## 2. Materials and Methods

**Figure 1:**
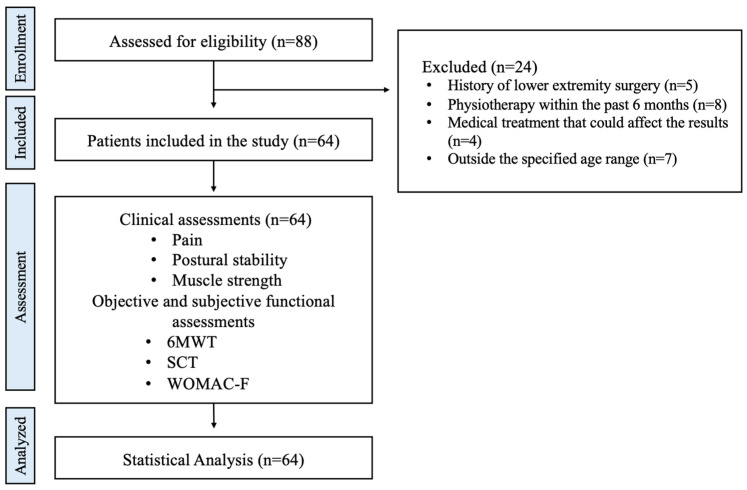
Flow diagram.

This research followed an observational design. The experimental workflow was organized into three primary stages: (1) enrollment and screening, where patients were assessed for eligibility and included after applying specific exclusion criteria; (2) assessment, comprising pain, postural stability, muscle strength, and objective and subjective functional performance; and (3) statistical analysis, where valid datasets were integrated into regression and mediation models (Figure 1).

### 2.1. Study Design and Protocol

This study was conducted in the Department of Physiotherapy and Rehabilitation, Faculty of Health Sciences, at Bezmialem Vakif University between August 2025 and February 2026. A consecutive sampling method was utilized to recruit patients with knee OA from the Outpatient Clinic of Physical Medicine and Rehabilitation, Bezmialem Vakif University Hospital, within this specified period. The study protocol was prospectively registered at ClinicalTrials.gov (Registration Number: NCT07066215). The study was conducted in accordance with the Declaration of Helsinki and approved by the local Institutional Ethics Committee. Written informed consent was obtained from all participants prior to participation.

Individuals diagnosed with bilateral primary knee OA according to the Kellgren–Lawrence classification (grades 2–3) were referred to the study by a specialist physician. Female participants aged 45 to 70 years, presenting with self-reported activity-related knee pain ≥3 on the visual analog scale (VAS) and the ability to ambulate independently, were eligible for inclusion. Individuals were excluded if they had a history of lower extremity surgery, malignancy, neuromuscular disease, vestibular disorders, or cardiopulmonary disease, or if they had received physiotherapy treatment or intra-articular injections within the previous six months. Following the confirmation of eligibility and receipt of informed consent, participants were scheduled for clinical assessments, including pain, postural stability, muscle strength, and functional performance. To ensure the accuracy of the measurements, participants were instructed to abstain from using analgesic medications on the day of the evaluation and the preceding 24 h.

### 2.2. Outcome Measures

Pain intensity was assessed using a visual analog scale (VAS), consisting of a 10 cm horizontal line ranging from “no pain” (0) to “worst imaginable pain”. Participants were instructed to mark the point on the line that best represented their current level of activity-related knee pain intensity [18]. Postural stability was assessed using the clinical test of sensory integration of balance (CTSIB) on the Biodex Balance System (Biodex Medical Systems Inc., Shirley, NY, USA). This protocol was specifically carried out to evaluate the sensory organization components of postural control by assessing how effectively the central nervous system integrates and prioritizes visual, somatosensory (proprioceptive), and vestibular inputs to maintain upright balance under modified environmental constraints [19]. The Biodex Balance System consists of a movable platform connected to a computer-based system that objectively evaluates postural stability. Data were captured using a static force plate system operating at a sampling rate of 20 Hz. The system utilizes integrated strain gauges to record the patient’s resultant center of pressure (COP) as sequential (X, Y) coordinate samples, which represent the projection of their center of gravity on the platform. Specifically, the system derives the sway angle relative to the center based on these (X, Y) coordinates and the estimated height of the patient’s COG, which is calculated using the manufacturer’s standardized biomechanical constant (0.55 X patient height). The primary outcome metric, the composite sway index (CSI), was calculated as the standard deviation of the patient’s stability index (i.e., the variance in COP displacement) during the test. Postural stability was evaluated under four different conditions: eyes open on a firm surface, eyes closed on a firm surface, eyes open on a foam surface, and eyes closed on a foam surface. Participants were instructed to stand on the platform without external support and maintain their position for 30 s in each condition, with a 10-s rest interval between trials. Lower CSI scores represent better postural stability, whereas higher scores represent greater postural sway, indicating increased unsteadiness during the test [20,21]. Lower extremity muscle strength was assessed using the five-times sit-to-stand test (FTSST). Participants were seated on a standard 43 cm high armless chair with their backs against the backrest and arms folded across the chest. Upon the command “go,” participants were instructed to stand up and sit down five times as quickly and safely as possible. The time required to complete the five repetitions was recorded in seconds using a calibrated digital manual stopwatch from the start command until the participant’s back contacted the backrest after the fifth repetition [22].

The Six-Minute Walk Test (6MWT), stair climbing test (SCT), and Western Ontario and McMaster Universities Osteoarthritis Index (WOMAC) were used to evaluate functionality. The SCT was performed on a flight of stairs consisting of nine steps with a step height of approximately 17 cm and equipped with a handrail. Participants were instructed to ascend and descend the stairs as quickly and safely as possible whilst wearing their usual footwear. The total time required to ascend and descend the stairs was recorded in seconds using a calibrated digital manual stopwatch by the same unblinded physical therapist [23]. A practice trial was conducted before the test to ensure that the participants were familiar with the procedure. The 6MWT was conducted according to standardized clinical protocols. Participants were instructed to walk as fast as possible along a 30 m corridor marked by two cones placed 30 m apart. The total distance walked during the 6 min period was quantified in meters by counting the completed laps along a 30 m indoor corridor explicitly demarcated with standardized floor markings, with any remaining fractional distance measured using a standard manual tape measure [24]. The WOMAC is a widely used and validated questionnaire designed to evaluate symptoms and functional limitations in individuals with knee and hip osteoarthritis. The questionnaire is divided into three subscales: pain, stiffness, and function. The function subscale (WOMAC-F), consisting of 17 items, was used for analysis, with higher scores indicating greater functional limitation. The Turkish version of the WOMAC was proven to be valid and reliable by Tüzün et al. [25]. Standardized rest intervals were provided between all tests and successive measurement trials, not only to restore baseline performance but also to minimize fatigue-related measurement bias across the entire assessment protocol. This approach is consistent with previous studies highlighting the importance of adequate recovery periods for obtaining reliable motor performance measurements [26,27].

### 2.3. Statistical Analysis

Statistical analyses were performed using IBM SPSS Version 25.0. The normality of the data distribution was assessed using the Shapiro–Wilk test. Descriptive and clinical characteristic statistics were presented as mean and standard deviation. Pearson’s correlation coefficients were used to examine the associations between all variables, including potential covariates (age, BMI, and pain). Prior to running the regression models, the assumptions of linearity and homoscedasticity were verified through residual diagnostics. The primary objective of identifying the independent predictors of functional outcomes was addressed using hierarchical linear regression analysis. Three separate models were constructed for each dependent variable: SCT, 6MWT, and the WOMAC-F. In Block 1, demographic and clinical characteristics (age, BMI, and VAS pain scores) were entered as control variables to account for their established influence on functional status. In Block 2, the main independent variables of interest—lower extremity muscle strength and postural stability—were added using the “Enter” method. The incremental contribution of the second block was evaluated using the R^2^ change (∆R^2^) and its associated *p*-value. To ensure the validity of the regression models, multicollinearity was assessed using the Variance Inflation Factor (VIF) and tolerance values; a VIF < 10 was considered acceptable. Durbin–Watson statistics were used to check for the independence of residuals. Additionally, for the 6MWT model where a mediation effect was suspected, a formal mediation analysis was conducted using the PROCESS macro (Model 4). The significance of the indirect effect was determined using bootstrapping with 5000 resamples; a 95% confidence interval (CI) that did not include zero was considered statistically significant. The level of significance for all tests was set at *p* < 0.05.

To assess sample adequacy, a post-hoc power analysis was performed for the primary outcome (6MWT) using G*Power 3.1. Based on the hierarchical regression model (f^2^ = 0.209, α = 0.05, two tested predictors, five total predictors, n = 64), the achieved statistical power was calculated as 0.88, indicating that the sample size was sufficient to detect the observed effect.

## 3. Results

A total of 88 female patients were initially screened for eligibility. Of these, 24 were excluded based on the predefined exclusion criteria (history of lower extremity surgery, physiotherapy within the past 6 months, medical treatment that could affect the results, and being outside of the specified age range). Consequently, 64 patients who met all inclusion criteria and provided informed consent were included in the final analysis. The mean age of the participants was 55.71 ± 5.99 years, and the mean BMI was 32.27 ± 4.82 kg/m^2^. Descriptive statistics regarding clinical scores and physical performance measures are summarized in Table 1.

For the SCT, the first block of the hierarchical regression analysis revealed that age (ß = 0.410, *p* < 0.010) and BMI (ß = 0.299, *p* = 0.008) were significant predictors (R^2^ = 0.308, Adj.R^2^ = 0.273, *p* < 0.001). The addition of the second block resulted in a significant increase in the explained variance (∆R^2^ = 0.103, *p* < 0.001). In the final model, age (ß = 0.248, *p* = 0.033), BMI (ß = 0.261, *p* = 0.014), and muscle strength (ß = 0.330, *p* = 0.005) remained significant independent predictors (R^2^ = 0.410, Adj.R^2^ = 0.360, *p* < 0.001) (Table 2).

For the 6MWT, the first block comprising age, BMI, and VAS scores significantly contributed to the model (R^2^ = 0.172, Adj.R^2^ = 0.131, *p* = 0.010). In this initial step, age (ß = −0.293, *p* = 0.016) and BMI (ß = −0.250, *p* = 0.039) were significant predictors. However, when muscle strength and postural stability were added in the second block, the model’s explanatory power increased significantly (∆R^2^ = 0.143, *p* < 0.001). In the final model, muscle strength emerged as the sole significant predictor (ß = −0.409, *p* = 0.001), while the effects of age and BMI became non-significant (Table 3). Correlations between independent variables and the potential mediator were examined; mediation analysis was subsequently conducted only for variables exhibiting a significant relationship (detailed correlation results are provided in Appendix A Table A1, and the corresponding scatter plots are presented in the Supplementary Material). The results indicated that muscle strength fully mediated the relationship between age and 6MWT performance. The total effect of age on the 6MWT (ß = −3.065, *p* = 0.009) became non-significant (ß = −1.249, *p* = 0.283) after controlling for muscle strength. The indirect effect of age on the 6MWT through muscle strength was statistically significant, as the 95% Bootstrap Confidence Interval did not cross zero [95% CI: −3.392, −0.481] (Table 4).

The hierarchical regression for the WOMAC-F showed that the first block explained 14.9% of the variance (R^2^ =0.189, Adj.R^2^ = 0.149, *p* = 0.005). In the final model, only the VAS (ß = 0.385, *p* = 0.001) and postural stability (ß = 0.243, *p* = 0.040) were identified as significant predictors. Notably, muscle strength did not significantly contribute to the subjective perception of functional difficulty (*p* = 0.245) (Table 5).

## 4. Discussion

Knee OA is a progressive and degenerative musculoskeletal disease that can impair functionality. Early identification of markers affecting functionality and the determination of appropriate treatment strategies are important in disease management. Studies indicate that compared to men, women with knee OA exhibit poorer physical performance, greater disease severity, and a more pronounced impact of muscle weakness on functional capacity [28,29,30]. The present study aimed to elucidate the independent contributions of muscle strength and postural stability to functional outcomes in women with bilateral knee OA whilst controlling for age, BMI, and pain. Our primary finding was that muscle strength is the most powerful predictor of objective performance-based tasks (SCT and 6MWT), even after accounting for demographic factors. In contrast, subjective functional status (WOMAC-F) was primarily driven by pain intensity and postural stability, suggesting a clear dissociation between how patients perform and how they perceive their limitations.

The stair climbing test and the Six-Minute Walk Test are among the performance-based measures recommended by the Osteoarthritis Research Society International (OARSI) as key indicators of physical function [17]. Previous studies have consistently demonstrated that reduced muscle strength in patients with knee OA is associated with impaired walking and stair climbing performance [28,31,32]. The results of the present study indicate that age, BMI, and muscle strength exert a significant impact on stair climbing and descending performance. Similarly, Bily et al. (2019) reported that knee extensor strength, evaluated via isometric and leg press measurements, was a high-level predictor of stair test performance in patients with advanced knee OA [33].

The five-times sit-to-stand test, a well-recognized method for assessing lower extremity strength and power, was utilized in this study [34]. Notably, while muscle strength emerged as the most potent predictor of SCT performance, age and BMI maintained their statistical significance within the final regression model. Unlike the 6MWT results, where the impact of age was fully mediated by strength, the sustained influence of age and BMI on stair climbing capacity suggests that these factors exert a more direct effect on high-demand functional tasks. It has been noted that age-related sarcopenia and obesity reduce physical performance, with obesity being specifically associated with poorer outcomes in elderly populations with OA [35,36,37]. This indicates that for activities requiring significant vertical displacement, factors beyond strength, such as age-related joint stiffness or the direct mechanical burden of increased BMI, independently contribute to functional limitations.

Furthermore, Casaña et al. (2021) [38] observed a significant relationship between stair climbing performance and isometric knee extension strength in advanced knee OA but noted that postural stability was not a major determinant for these tasks. It has been reported that stair climbing performance is more influenced by dynamic balance tasks rather than static balance [38]. Consistent with this, the current study demonstrated that, while lower extremity strength was a significant predictor for the SCT, postural stability showed no significant association. In evaluating postural stability, the clinical test of sensory integration of balance (CTSIB) was employed across varying surfaces (firm/foam) and visual conditions (eyes open/closed). The composite sway index represents the individual’s overall postural sway performance, serving as a direct indicator of their postural stability under sensory challenges. Our results suggest that for activities focusing on dynamic force transfer and dynamic stabilization, the predictive value of sensory integration tests may remain limited, reflecting the patient’s fundamental perception of balance rather than their actual functional performance capacity.

The literature indicates that 6MWT performance is influenced not only by disease pathology but also by demographic and psychosocial factors [39,40]. In the current study, the first block of the hierarchical regression model for the 6MWT identified age and BMI as significant determinants; however, upon the inclusion of muscle strength in the second block, the predictive power of these variables diminished, and muscle strength emerged as the sole significant predictor. Mediation analysis further revealed that muscle strength serves as a full mediator in the relationship between age and walking capacity. This finding supports the premise that muscle weakness resulting from motor unit loss and reduced muscle mass associated with the aging process is primarily responsible for the decline in walking capacity [41,42].

Regarding BMI, a similar mediatory role was not observed. This suggests that while a high BMI negatively affects muscle quality [43], it may also restrict performance through a direct increase in mechanical loading on the joints. In this context, our results emphasize that focusing directly on muscle strength is a critical strategy to compensate for functional limitations imposed by non-modifiable factors such as aging or factors requiring long-term management like BMI. Although some studies in the literature suggest that pain may also be a predictor of 6MWT distance [44], no significant association between pain and walking distance was detected in the present study.

The WOMAC is a widely utilized, patient-reported outcome measure with demonstrated reliability and validity for assessing functional status in knee OA [45]. Our findings indicate that pain and postural stability are significant determinants of WOMAC-F scores. Notably, the emergence of balance and pain rather than muscle strength as significant predictors for the WOMAC function subscale suggests that patients are influenced by mechanisms distinct from those measured by objective performance tests when evaluating their own functional limitations. This divergence highlights that pain in knee OA is a multidimensional experience not only driven by peripheral structural damage, but also heavily influenced by central pain-processing mechanisms and psychosocial interactions [46]. In particular, central sensitization can amplify nociceptive signaling at the brain level, leading to heightened pain perception and secondary motor impairments even in mild radiographic stages [47,48]. Consequently, self-reported metrics like the WOMAC function subscale profoundly reflect these central nervous system processes and emotional responses, whereas objective performance-based assessments capture raw actual functional capacity. This phenomenon can be explained by the marked dissociation between what patients can actually achieve and what they perceive they can achieve [16]. In the literature, it is stated that the presence of chronic pain may lead patients to perceive themselves as more limited, even if it does not directly decrease their performance capacity [49].

Maly et al. (2006) reported that a substantial portion of the variance in the WOMAC physical function subscale (68.8%) is explained by pain alone, suggesting that performance measures may not fully capture a patient’s functional perception in daily life [50]. Our findings, where pain and postural stability emerged as primary factors affecting WOMAC scores rather than strength, support the premise that patients focus more on their pain levels and sense of stability rather than their mechanical capacity when reporting functional limitations. Progressive joint degeneration in knee OA compromises articular mechanoreceptors, leading to impaired proprioception and sensory integration deficits, which can adversely affect postural stability and movement confidence. Reduced quality of afferent input from the knee joint may alter central sensorimotor processing, leading individuals to perceive daily activities as more challenging or unstable [51,52]. Postural instability may increase concerns about losing balance, falling, or experiencing pain during movement. As the WOMAC function subscale reflects patients’ perceptions of difficulty during daily activities, these perceptions of instability may contribute to higher levels of self-reported disability independent of actual physical performance. This complex interplay between balance deficits, kinesiophobia, and perceived functional status has been well established in the literature, demonstrating that impaired postural control is directly associated with heightened fear avoidance beliefs, catastrophizing, and severe self-reported functional limitations in patients with knee osteoarthritis [9,53]. In contrast to our results, Sanchez-Ramirez (2013) stated that postural control is associated with activity limitation and is only significant for performance-based measures, with no significant findings for self-reported activities [54]. Conversely, our findings regarding muscle strength differ from certain meta-analyses in the literature. For instance, Culvenor et al. (2017) identified reduced knee extensor strength as a primary risk factor for poor WOMAC scores [55]. However, the dominance of pain over self-reported measures in the present study aligns with other research, suggesting that pain levels may overshadow physical capacity and heavily influence functional reporting [56,57].

In a study investigating the determinants of self-reported versus performance-based physical function, Wang et al. (2024) reported that while pain and muscle strength were predictors for self-reported function (with pain being the primary determinant), muscle strength was the fundamental predictor for performance-based outcomes [16]. Similarly, Abujaber et al. (2024) noted that in patients with advanced knee OA awaiting total knee arthroplasty, pain was associated with the subjective aspect of physical function, whereas strength was linked to both perceived and actual functional capacity [58]. In our study, functional performance was evaluated using the SCT and 6MWT, with muscle strength identified as the primary determinant. In contrast, no significant relationship was found between muscle strength and the WOMAC function scale; instead, pain and postural stability emerged as the key predictors.

Although these conventional performance-based assessments are highly reliable, the repetitive nature of consecutive physical trials across sessions may occasionally induce task monotonicity, potentially affecting optimal participant engagement. To address this in future research, clinical protocols could benefit from integrating immersive virtual reality (VR) environments or gamified biofeedback systems to sustain active motivation [59,60]. Furthermore, while the 6MWT, FTSST, and SCT provide clinically meaningful and widely accepted indicators of functional performance, they do not directly quantify detailed joint kinematics such as angular velocity, segment orientation, or movement trajectories. Biomechanically, these parameters may provide additional insight into compensatory movement strategies in individuals with bilateral knee osteoarthritis. Recent advances in wearable inertial measurement units (IMUs) and motion-tracking technologies have made detailed kinematic assessment increasingly feasible in rehabilitation research [61,62]. Although the present study intentionally focused on clinically accessible and widely applicable outcome measures, future studies incorporating such technologies may contribute to a more comprehensive biomechanical understanding of functional limitations in knee osteoarthritis.

Consequently, while muscle strength forms the foundation of physical capacity, effective pain management and the improvement of postural control may be vital in enhancing a patient’s perception of their own functionality and quality of life. Particularly for female patients, focusing on balance control in addition to pain management and lower extremity strength training is of critical importance for optimizing functional outcomes.

The present study has several strengths. First, both performance-based and patient-reported functional outcomes were evaluated simultaneously, allowing a comprehensive assessment of functional status in women with bilateral knee osteoarthritis. Second, the independent contributions of muscle strength and postural stability were examined after controlling for important confounding factors, including age, BMI, and pain. Third, the use of hierarchical regression and mediation analyses provided a more detailed understanding of the mechanisms underlying functional performance and perceived disability.

### Limitations

The present study has several limitations that should be considered when interpreting the findings. First, the study population consisted exclusively of female participants, which limits the generalizability of the results to male patients with knee OA. Second, the mean age of the participants was relatively young compared to typical knee OA populations, potentially influencing the functional performance and strength levels observed. The methodological approach of the current study relied on standard computerized postural stability testing. Future studies may incorporate immersive virtual reality paradigms for capturing dynamic motor adaptation in highly engaging environments in knee osteoarthritis. Finally, the study did not include patients with advanced-stage (K-L Grade IV) knee OA. Future research involving a more diverse cohort regarding gender, a broader age range specifically targeting elderly populations, and all stages of OA severity is warranted to validate these results.

## 5. Conclusions

This study highlights that muscle strength is the primary determinant of objective functional performance, specifically in walking and stair climbing tasks, among female patients with knee osteoarthritis. Importantly, our mediation analysis reinforces these findings by revealing that muscle strength serves as a full mediator in the relationship between age and walking capacity, demonstrating that age-related declines in objective performance are primarily driven by the progressive loss of muscle capacity rather than chronological age alone. Furthermore, the observed dissociation between objective performance and patient-reported outcomes suggests that while mechanical capacity drives physical tasks, factors such as pain and postural stability are more influential in shaping a patient’s subjective perception of their function. This clear distinction emphasizes that performance-based tests and self-reported metrics capture fundamentally distinct clinical dimensions, heavily driven by peripheral mechanical factors and centralized sensorimotor and psychosocial processes, respectively. From a clinical perspective, these results underscore that focusing on lower extremity strength training is an effective strategy to compensate for functional limitations. Additionally, integrating pain management and balance control into rehabilitation programs, particularly for women, may be essential to improve both actual functional capacity and the patient’s perceived quality of life. Future longitudinal studies with more diverse populations, including advanced age groups, are needed to further explore these mediation pathways across all stages of OA severity.

## Figures and Tables

**Table 1:** Demographic and clinical characteristics of participants (n = 64).

Variables	Mean ± Sd (Min–Max)
Age (years)	55.71 ± 5.99 (45–65)
BMI (kg/m^2^)	32.27 ± 4.82 (21.5–39.8)
VAS	6.56 ± 2.03 (3–10)
FTSST (s)	11.85 ± 2.44 (6.90–18.30)
CTSIB (CSI)	1.48 ± 0.30 (0.90–2.30)
SCT (s)	12.42 ± 2.45 (7.65–19.24)
6MWT (m)	491.85 ± 56.88 (322–615)
WOMAC-F	22.70 ± 9.99 (5–44)
K-L Grade II/III n (%)	35(54.7%)/29(45.3%) (II–III)

BMI: body mass index; VAS: visual analog scale; FTSST: five-times sit-to-stand test; CTSIB: clinical test of sensory integration of balance; CSI: composite sway index; SCT: stair climbing test; 6MWT: Six-Minute Walk Test; WOMAC: Western Ontario and McMaster Universities Osteoarthritis Index; K-L: Kellgren–Lawrence.

**Table 2:** Hierarchical regression analysis of predictors for the stair climbing test (SCT).

SCT		B	SE	β	*p*
Block 1	Age	0.168	0.044	0.410	**<0.001**
	BMI	0.152	0.055	0.299	**0.008**
	VAS	0.150	0.130	0.125	0.252
Model summary-1		R^2^ = 0.308	Adj R^2^ = 0.273		***p* < 0.001**
		**B**	**SE**	**β**	***p***
Block 2	Age	0.101	0.046	0.248	**0.033**
	BMI	0.133	0.052	0.261	**0.014**
	VAS	0.114	0.123	0.094	0.359
	FTSST	0.331	0.113	0.330	**0.005**
	CTSIB	1.028	0.844	0.126	0.228
Model summary-2		R^2^ = 0.410	Adj R^2^ = 0.360	ΔR^2^ = 0.103	***p* < 0.001**

BMI: body mass index; VAS: visual analog scale; FTSST: five-times sit-to-stand test; CTSIB: clinical test of sensory integration of balance. Note: Bold values indicate statistically significant result.

**Table 3:** Hierarchical regression analysis of predictors for Six-Minute Walk Test (6MWT).

6MWT		B	SE	β	*p*
Block 1	Age	−2.778	1.123	−0.293	**0.016**
	BMI	−2.952	1.398	−0.250	**0.039**
	VAS	−1.798	3.297	−0.064	0.587
Model summary-1		R^2^ = 0.172	Adj R^2^ = 0.131		***p* = 0.010**
		**B**	**SE**	**β**	***p***
Block 2	Age	−0.973	1.162	−0.103	0.406
	BMI	−2.395	1.304	−0.203	0.071
	VAS	−0.905	3.071	−0.032	0.769
	FTSST	−9.522	2.836	−0.409	**0.001**
	CTSIB	−18.947	21.100	−0.100	0.373
Model summary-2		R^2^ = 0.316	Adj R^2^ = 0.257	ΔR^2^ = 0.143	***p* < 0.001**

BMI: body mass index; VAS: visual analog scale; FTSST: five-times sit-to-stand test; CTSIB: clinical test of sensory integration of balance. Note: Bold values indicate statistically significant result.

**Table 4:** Mediation analysis of muscle strength in the relationship between age and 6MWT performance.

	ß	SE	t	*p*	LLCI	ULCI
Total effect	−3.065	1.140	−2.688	**0.009**	−5.345	−0.323
Direct effect						
Age	−1.249	1.155	−1.081	0.283	−3.559	1.060
Indirect effect						
Age–FTSST	−1.816	0.758			**−3.392**	**−0.481**

FTSST: five-times sit-to-stand test; SE: standard error; LLCI: lower-limit confidence interval; ULCI: upper-limit confidence interval. Note: Bold values indicate statistically significant result.

**Table 5:** Hierarchical regression analysis of predictors for WOMAC-F.

WOMAC-F		B	SE	β	*p*
Block 1	Age	−0.048	0.195	−0.029	0.805
	BMI	0.204	0.243	0.098	0.405
	VAS	2.055	0.573	0.418	**0.001**
Model summary-1		R^2^ = 0.189	Adj R^2^ = 0.149		***p* = 0.005**
		**B**	**SE**	**β**	***p***
Block 2	Age	−0.231	0.212	−0.139	0.279
	BMI	0.174	0.238	0.084	0.466
	VAS	1.894	0.560	0.385	**0.001**
	FTSST	0.607	0.517	0.148	0.245
	CTSIB	8.085	3.845	0.243	**0.040**
Model summary-2		R^2^ = 0.264	Adj R^2^ = 0.200	Δ R^2^ = 0.074	***p* = 0.003**

BMI: body mass index; VAS: visual analog scale; FTSST: five-times sit-to-stand test; CTSIB: clinical test of sensory integration of balance; WOMAC: Western Ontario and McMaster Universities Osteoarthritis Index. Note: Bold values indicate statistically significant result.

## Data Availability

The raw data supporting the conclusions of this article will be made available by the authors upon request.

## Supplementary Materials

The following supporting information can be downloaded at: https://www.mdpi.com/article/10.3390/healthcare14131880/s1, Figure S1: Correlation between age and the Six-Minute Walk Test (6MWT) distance; Figure S2: Correlation between BMI and 6MWT distance; Figure S3: Correlation between lower extremity muscle strength (FTSST) and 6MWT distance; Figure S4: Correlation between FTSST and age; Figure S5: Correlation between age and stair climbing test (SCT); Figure S6: Correlation between BMI and SCT; Figure S7: Correlation between FTSST and SCT; Figure S8: Correlation between VAS and WOMAC-F; Figure S9: Correlation between CTSIB and WOMAC-F.

## Abbreviations

The following abbreviations are used in this manuscript:

OA	Osteoarthritis
FTSST	Five-Times Sit-to-Stand Test
6MWT	Six-Minute Walk Test
SCT	Stair Climbing Test
BMI	Body Mass Index
VAS	Visual Analog Scale
CTSIB	Clinical Test of Sensory Integration of Balance
WOMAC	Western Ontario and McMaster Universities Osteoarthritis Index
OARSI	Osteoarthritis Research Society International

## Appendix A

**Table A1:** Pearson’s coefficients (r) of demographic and clinical parameters.

	Age (Years)	BMI (kg/m^2^)	VAS	FTSST (s)	CTSIB (SI)	6MWT (m)	SCT (s)	WOMAC-F
Age (years)	-							
BMI (kg/m^2^)	0.121	-						
VAS	0.001	0.062	-					
FTSST (s)	**0.432 ****	0.173	0.061	-				
CTSIB (CSI)	0.195	0.016	0.102	0.101	-			
6MWT (m)	**−0.323 ****	**−0.290 ***	−0.080	**−0.500 ****	−0.168	-		
SCT (s)	**0.447 ****	**0.356 ****	0.144	**0.501 ****	0.221	**−0.575 ****	-	
WOMAC-F	−0.017	0.121	**0.424 ****	0.151	**0.271 ***	−0.160	**0.274 ***	

BMI: body mass index; VAS: visual analog scale; FTSST: five-times sit-to-stand test; CTSIB: clinical test of sensory integration of balance; CSI: composite sway index; SCT: stair climbing test; 6MWT: Six-Minute Walk Test; WOMAC: Western Ontario and McMaster Universities Osteoarthritis Index. Significant correlations in bold. ** *p* < 0.01; * *p* < 0.05.

## References

[1] Goldring M.B., Goldring S.R. (2007). Osteoarthritis. J. Cell. Physiol..

[2] Cihan E., Şahbaz Pirinççi C., Leblebicier M.A. (2024). Effects of osteoarthritis grades on pain, function and quality of life. J. Back Musculoskelet. Rehabil..

[3] Muraki S., Akune T., Teraguchi M., Kagotani R., Asai Y., Yoshida M., Tokimura F., Tanaka S., Oka H., Kawaguchi H. (2015). Quadriceps muscle strength, radiographic knee osteoarthritis and knee pain: The ROAD study. BMC Musculoskelet. Disord..

[4] Bennell K.L., Hunt M.A., Wrigley T.V., Lim B.W., Hinman R.S. (2008). Role of Muscle in the Genesis and Management of Knee Osteoarthritis. Rheum. Dis. Clin. N. Am..

[5] Patterson B.E., Girdwood M.A., West T.J., Bruder A.M., Øiestad B.E., Juhl C., Culvenor A.G. (2023). Muscle strength and osteoarthritis of the knee: A systematic review and meta-analysis of longitudinal studies. Skelet. Radiol..

[6] Hinman R.S., Hunt M.A., Creaby M.W., Wrigley T.V., McManus F.J., Bennell K.L. (2010). Hip muscle weakness in individuals with medial knee osteoarthritis. Arthritis Care Res..

[7] Petrella M., Neves T.M., Reis J.G., Gomes M.M., Oliveira R.D., Abreu D.C. (2012). Postural control parameters in elderly female fallers and non-fallers diagnosed or not with knee osteoarthritis. Rev. Bras. Reumatol..

[8] Liu C., Wan Q., Zhou W., Feng X., Shang S. (2017). Factors associated with balance function in patients with knee osteoarthritis: An integrative review. Int. J. Nurs. Sci..

[9] Sánchez-Herán Á., Agudo-Carmona D., Ferrer-Peña R., López-de-Uralde-Villanueva I., Gil-Martínez A., Paris-Alemany A., La Touche R. (2016). Postural Stability in Osteoarthritis of the Knee and Hip: Analysis of Association With Pain Catastrophizing and Fear-Avoidance Beliefs. PM&R.

[10] Hurley M.V., Scott D.L., Rees J., Newham D.J. (1997). Sensorimotor changes and functional performance in patients with knee osteoarthritis. Ann. Rheum. Dis..

[11] Turcot K., Armand S., Fritschy D., Hoffmeyer P., Suva D. (2012). Sit-to-stand alterations in advanced knee osteoarthritis. Gait Posture.

[12] Tan J.S., Tikoft E., O’Sullivan P., Smith A., Campbell A., Caneiro J.P., Kent P. (2021). The Relationship Between Changes in Movement and Activity Limitation or Pain in People With Knee Osteoarthritis: A Systematic Review. J. Orthop. Sports Phys. Ther..

[13] White D.K., Master H. (2016). Patient-Reported Measures of Physical Function in Knee Osteoarthritis. Rheum. Dis. Clin. N. Am..

[14] Alnahdi A.H., Zeni J.A., Snyder-Mackler L. (2012). Muscle impairments in patients with knee osteoarthritis. Sports Health.

[15] Knoop J., Steultjens M.P., van der Leeden M., van der Esch M., Thorstensson C.A., Roorda L.D., Lems W., Dekker J. (2011). Proprioception in knee osteoarthritis: A narrative review. Osteoarthr. Cartil..

[16] Wang Q.W., Man G.C., Choi B.C., Yeung Y.M., Qiu J.H., Lu X.M., Ong M.T., Yung P.S. (2024). The predictors to self-reported and performance-based physical function in knee osteoarthritis patients: A cross-sectional study. Front. Cell Dev. Biol..

[17] Dobson F., Hinman R.S., Hall M., Terwee C.B., Roos E.M., Bennell K.L. (2012). Measurement properties of performance-based measures to assess physical function in hip and knee osteoarthritis: A systematic review. Osteoarthr. Cartil..

[18] Alghadir A.H., Anwer S., Iqbal A., Iqbal Z.A. (2018). Test-retest reliability, validity, and minimum detectable change of visual analog, numerical rating, and verbal rating scales for measurement of osteoarthritic knee pain. J. Pain Res..

[19] Shumway-Cook A., Horak F.B. (1986). Assessing the Influence of Sensory Interaction on Balance: Suggestion from the Field. Phys. Ther..

[20] Bıodex Balance System Sd Operatıon & Servıce Manual. https://data2.manualslib.com/cpdf/37/185/18463/18665c.pdf.

[21] Cachupe W.J.C., Shifflett B., Kahanov L., Wughalter E.H. (2001). Reliability of Biodex Balance System Measures. Meas. Phys. Educ. Exerc. Sci..

[22] Khuna L., Soison T., Plukwongchuen T., Tangadulrat N. (2024). Reliability and concurrent validity of 30-s and 5-time sit-to-stand tests in older adults with knee osteoarthritis. Clin. Rheumatol..

[23] Dobson F., Hinman R.S., Roos E.M., Abbott J.H., Stratford P., Davis A.M., Buchbinder R., Snyder-Mackler L., Henrotin Y., Thumboo J. (2013). OARSI recommended performance-based tests to assess physical function in people diagnosed with hip or knee osteoarthritis. Osteoarthr. Cartil..

[24] Dobson F., Hinman R.S., Hall M., Marshall C.J., Sayer T., Anderson C., Newcomb N., Stratford P.W., Bennell K.L. (2017). Reliability and measurement error of the Osteoarthritis Research Society International (OARSI) recommended performance-based tests of physical function in people with hip and knee osteoarthritis. Osteoarthr. Cartil..

[25] Tüzün E.H., Eker L., Aytar A., Daşkapan A., Bayramoğlu M. (2005). Acceptability, reliability, validity and responsiveness of the Turkish version of WOMAC osteoarthritis index. Osteoarthr. Cartil..

[26] Suglia V., Camardella C., Rinaldi G., Chiaradia D., Buongiorno D., Leonardis D., Zhou H., Frisoli A., Bevilacqua V. (2026). Muscle networks analysis on an active occupational shoulder exoskeleton. Biomed. Signal Process..

[27] Rinaldi G., Suglia V., Tiseni L., Camardella C., Xiloyannis M., Masia L., Buongiorno D., Bevilacqua V., Frisoli A., Chiaradia D. (2026). Towards a healthier workplace: How Flexos, an active and bilateral shoulder exoskeleton, provides support in weight-lifting and carrying tasks. IEEE Trans. Robot..

[28] Bacon K.L., Segal N.A., Øiestad B.E., Lewis C.E., Nevitt M.C., Brown C., Felson D.T. (2019). Concurrent Change in Quadriceps Strength and Physical Function Over Five Years in the Multicenter Osteoarthritis Study. Arthritis Care Res..

[29] Logerstedt D.S., Zeni J., Snyder-Mackler L. (2014). Sex differences in patients with different stages of knee osteoarthritis. Arch. Phys. Med. Rehabil..

[30] Srikanth V.K., Fryer J.L., Zhai G., Winzenberg T.M., Hosmer D., Jones G. (2005). A meta-analysis of sex differences prevalence, incidence and severity of osteoarthritis. Osteoarthr. Cartil..

[31] Accettura A.J., Brenneman E.C., Stratford P.W., Maly M.R. (2015). Knee Extensor Power Relates to Mobility Performance in People With Knee Osteoarthritis: Cross-Sectional Analysis. Phys. Ther..

[32] van der Esch M., Holla J.F., van der Leeden M., Knol D.L., Lems W.F., Roorda L.D., Dekker J. (2014). Decrease of muscle strength is associated with increase of activity limitations in early knee osteoarthritis: 3-year results from the cohort hip and cohort knee study. Arch. Phys. Med. Rehabil..

[33] Bily W., Sarabon N., Löfler S., Franz C., Wakolbinger R., Kern H. (2019). Relationship Between Strength Parameters and Functional Performance Tests in Patients With Severe Knee Osteoarthritis. PM&R.

[34] Porto J., Peres-Ueno M., de Matos Brunelli Braghin R., Scudilio G., de Abreu D. (2023). Diagnostic accuracy of the five times stand-to-sit test for the screening of global muscle weakness in community-dwelling older women. Exp. Gerontol..

[35] Sartorio A., Proietti M., Marinone P.G., Agosti F., Adorni F., Lafortuna C.L. (2004). Influence of gender, age and BMI on lower limb muscular power output in a large population of obese men and women. Int. J. Obes. Relat. Metab. Disord..

[36] Vincent H.K., Vincent K.R., Lamb K.M. (2010). Obesity and mobility disability in the older adult. Obes. Rev..

[37] Batsis J.A., Zbehlik A.J., Barre L.K., Bynum J.P.W., Pidgeon D., Bartels S.J. (2015). Impact of obesity on disability, function, and physical activity: Data from the Osteoarthritis Initiative. Scand. J. Rheumatol..

[38] Casaña J., Calatayud J., Silvestre A., Sánchez-Frutos J., Andersen L.L., Jakobsen M.D., Ezzatvar Y., Alakhdar Y. (2021). Knee extensor muscle strength is more important than postural balance for stair-climbing ability in elderly patients with severe knee osteoarthritis. Int. J. Environ. Res. Public Health.

[39] Capodaglio P., De Souza S.A., Parisio C., Precilios H., Vismara L., Cimolin V., Brunani A. (2013). Reference values for the 6-Min Walking Test in obese subjects. Disabil. Rehabil..

[40] Troosters T., Gosselink R., Decramer M. (1999). Six minute walking distance in healthy elderly subjects. Eur. Respir. J..

[41] Kaya R.D., Nakazawa M., Hoffman R.L., Clark B.C. (2013). Interrelationship between muscle strength, motor units, and aging. Exp. Gerontol..

[42] Hayashida I., Tanimoto Y., Takahashi Y., Kusabiraki T., Tamaki J. (2014). Correlation between Muscle Strength and Muscle Mass, and Their Association with Walking Speed, in Community-Dwelling Elderly Japanese Individuals. PLoS ONE.

[43] De Stefano F., Zambon S., Giacometti L., Sergi G., Corti M.C., Manzato E., Busetto L. (2015). Obesity, muscular strength, muscle composition and physical performance in an elderly population. J. Nutr. Health Aging.

[44] Yázigi F., Espanha M., Marques A., Teles J., Teixeira P. (2018). Predictors of walking capacity in obese adults with knee osteoarthritis. Acta Reumatol. Port..

[45] McConnell S., Kolopack P., Davis A.M. (2001). The Western Ontario and McMaster Universities Osteoarthritis Index (WOMAC): A review of its utility and measurement properties. Arthritis Care Res..

[46] Iijima H., Aoyama T., Fukutani N., Isho T., Yamamoto Y., Hiraoka M., Miyanobu K., Jinnouchi M., Kaneda E., Kuroki H. (2018). Psychological health is associated with knee pain and physical function in patients with knee osteoarthritis: An exploratory cross-sectional study. BMC Psychol..

[47] Finan P.H., Buenaver L.F., Bounds S.C., Hussain S., Park R.J., Haque U.J., Campbell C.M., Haythornthwaite J.A., Edwards R.R., Smith M.T. (2013). Discordance between pain and radiographic severity in knee osteoarthritis: Findings from quantitative sensory testing of central sensitization. Arthritis Rheum..

[48] Lluch E., Nijs J., Courtney C.A., Rebbeck T., Wylde V., Baert I., Wideman T.H., Howells N., Skou S.T. (2018). Clinical descriptors for the recognition of central sensitization pain in patients with knee osteoarthritis. Disabil. Rehabil..

[49] Wilfong J.M., Badley E.M., Power J.D., Gandhi R., Rampersaud Y.R., Perruccio A.V. (2020). Discordance between self-reported and performance-based function among knee osteoarthritis surgical patients: Variations by sex and obesity. PLoS ONE.

[50] Maly M.R., Costigan P.A., Olney S.J. (2006). Determinants of Self-Report Outcome Measures in People with Knee Osteoarthritis. Arch. Phys. Med. Rehabil..

[51] Sharma L. (1999). Proprioceptive Impairment In Knee Osteoarthritis. Rheum. Dis. Clin. N. Am..

[52] Khalaj N., Abu Osman N.A., Mokhtar A.H., Mehdikhani M., Wan Abas W.A. (2014). Balance and risk of fall in individuals with bilateral mild and moderate knee osteoarthritis. PLoS ONE.

[53] Ata A.M., Kara O., Tuncer B., Mermerci B. (2025). Association of balance with kinesiophobia and physical function in knee osteoarthritis. J. Bodyw. Mov. Ther..

[54] Sanchez-Ramirez D.C., van der Leeden M., Knol D.L., van der Esch M., Roorda L.D., Verschueren S., van Dieën J., Lems W.F., Dekker J. (2013). Association of postural control with muscle strength, proprioception, self-reported knee instability and activity limitations in patients with knee osteoarthritis. J. Rehabil. Med..

[55] Culvenor A.G., Ruhdorfer A., Juhl C., Eckstein F., Øiestad B.E. (2017). Knee Extensor Strength and Risk of Structural, Symptomatic, and Functional Decline in Knee Osteoarthritis: A Systematic Review and Meta-Analysis. Arthritis Care Res..

[56] Harrison A.L. (2004). The influence of pathology, pain, balance, and self-efficacy on function in women with osteoarthritis of the knee. Phys. Ther..

[57] Terwee C.B., van der Slikke R.M., van Lummel R.C., Benink R.J., Meijers W.G., de Vet H.C. (2006). Self-reported physical functioning was more influenced by pain than performance-based physical functioning in knee-osteoarthritis patients. J. Clin. Epidemiol..

[58] Abujaber S., Altubasi I., Hamdan M., Al-Zaben R., Bani-Ahmad O. (2024). Physical functioning in patients with end-stage knee osteoarthritis: A cross-sectional study in Jordan using self-reported questionnaire and performance-based tests. J. Back Musculoskelet. Rehabil..

[59] Martelli D., Xia B., Prado A., Agrawal S.K. (2019). Gait adaptations during overground walking and multidirectional oscillations of the visual field in a virtual reality headset. Gait Posture.

[60] Labruyère R., Gerber C.N., Birrer-Brütsch K., Meyer-Heim A., van Hedel H.J.A. (2013). Requirements for and impact of a serious game for neuro-pediatric robot-assisted gait training. Res. Dev. Disabil..

[61] Palazzo L., Suglia V., Grieco S., Buongiorno D., Brunetti A., Carnimeo L., Amitrano F., Coccia A., Pagano G., D’addio G. (2025). A Deep Learning-Based Framework Oriented to Pathological Gait Recognition with Inertial Sensors. Sensors.

[62] Tan J.S., Beheshti B.K., Binnie T., Davey P., Caneiro J.P., Kent P., Smith A., O’sullivan P., Campbell A. (2021). Human Activity Recognition for People with Knee Osteoarthritis—A Proof-of-Concept. Sensors.

