# Supplementary material for: Distinct Roles of Muscle Strength and Postural Stability in Objective and Subjective Function in Women with Bilateral Knee Osteoarthritis
Source: Healthcare (Basel). 2026 Jun 27;14(13):1880. doi: 10.3390/healthcare14131880 (PMC13362523; doi:10.3390/healthcare14131880)
Supplement: Supplementary file 1 [file healthcare-14-01880-s001.zip › healthcare-4366390-supplementary.pdf]

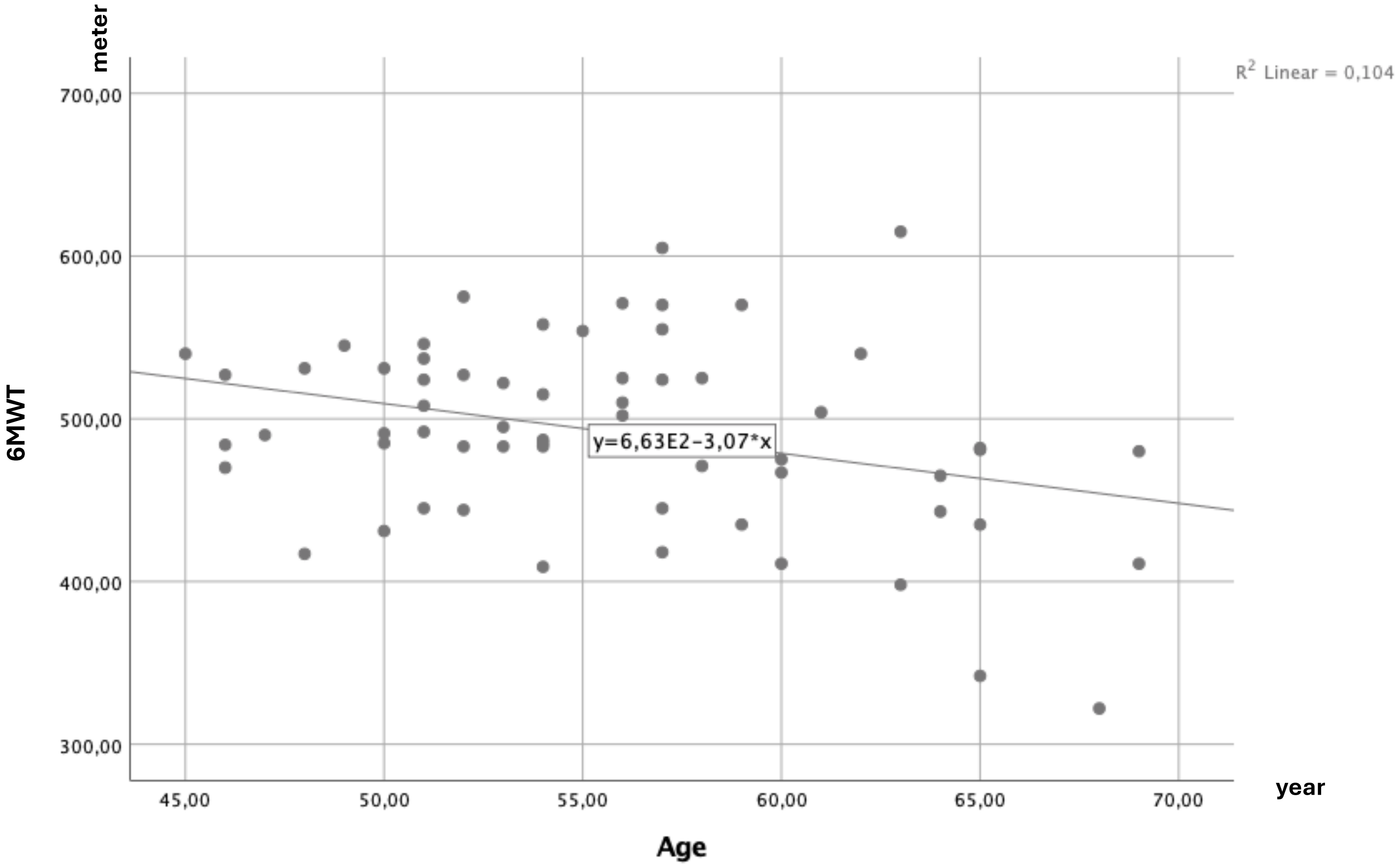

Figure S1: Correlation between age and the 6-Minute Walk Test (6MWT) distance (r=-0.323, p=0.009)

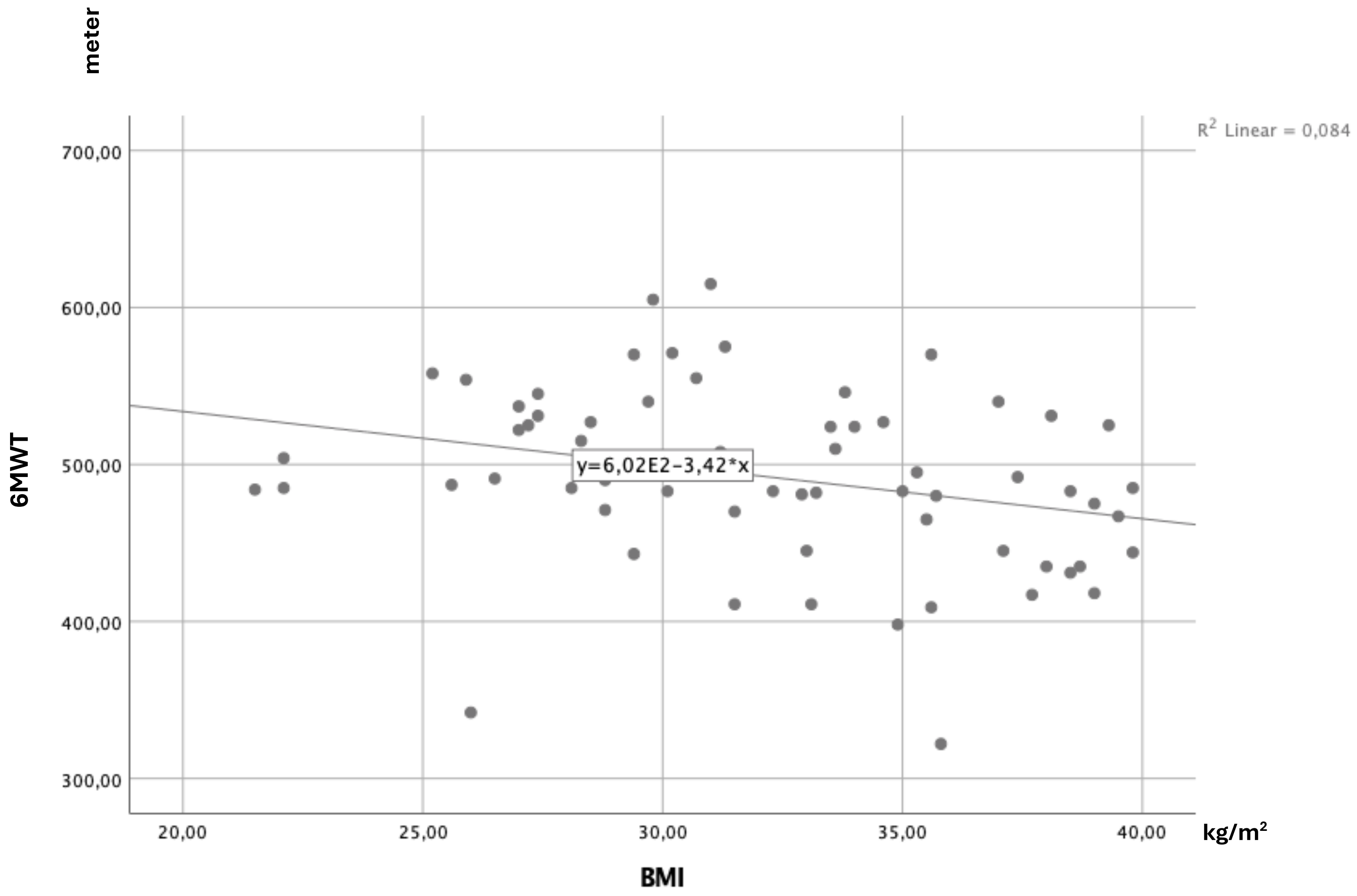

Figure S2: Correlation between BMI and 6MWT distance (r= -0.290, p=0,020)

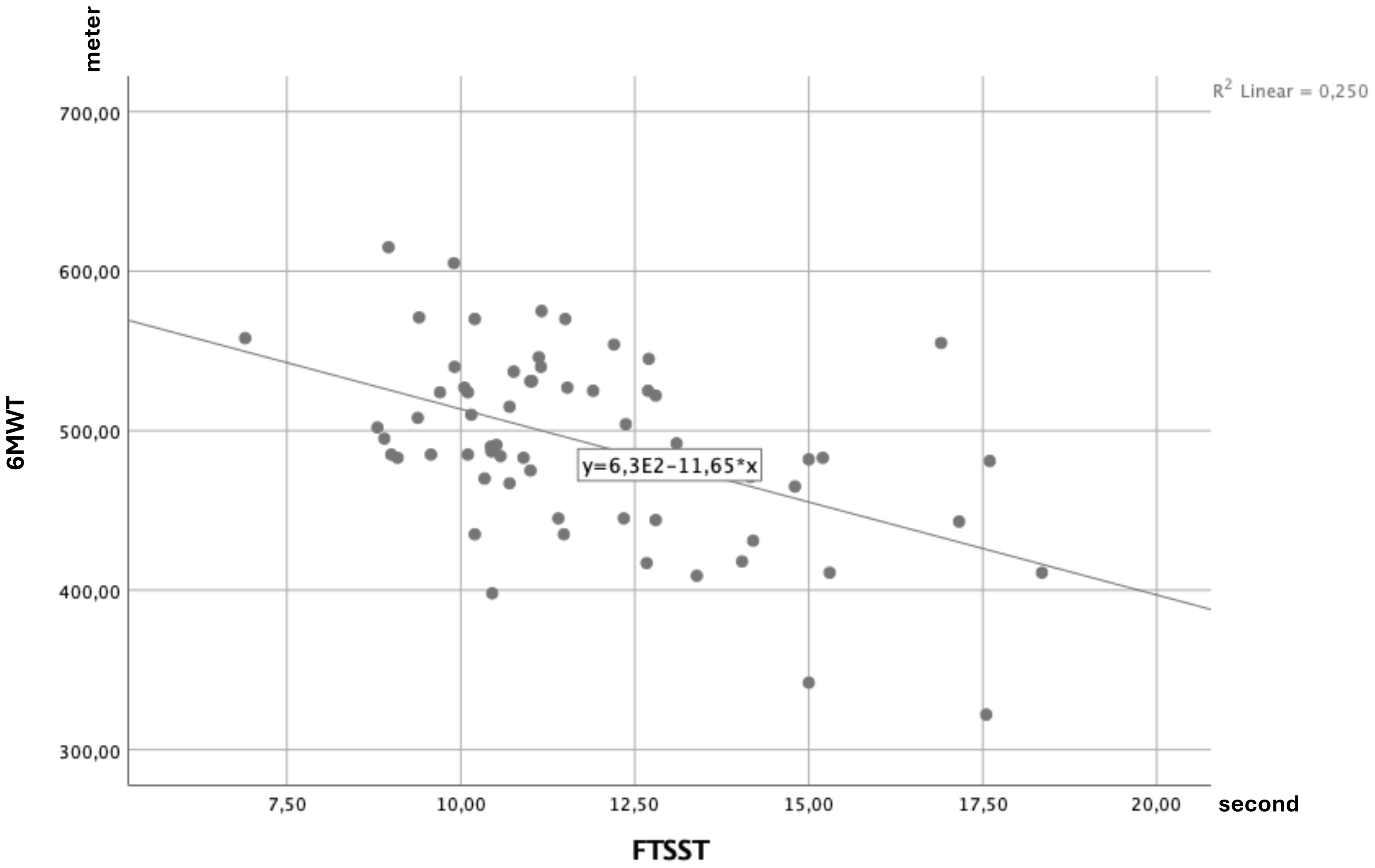

Figure S3: Correlation between lower extremity muscle strength (FTSST) and 6MWT distance (r=-0.500, p <0.001)

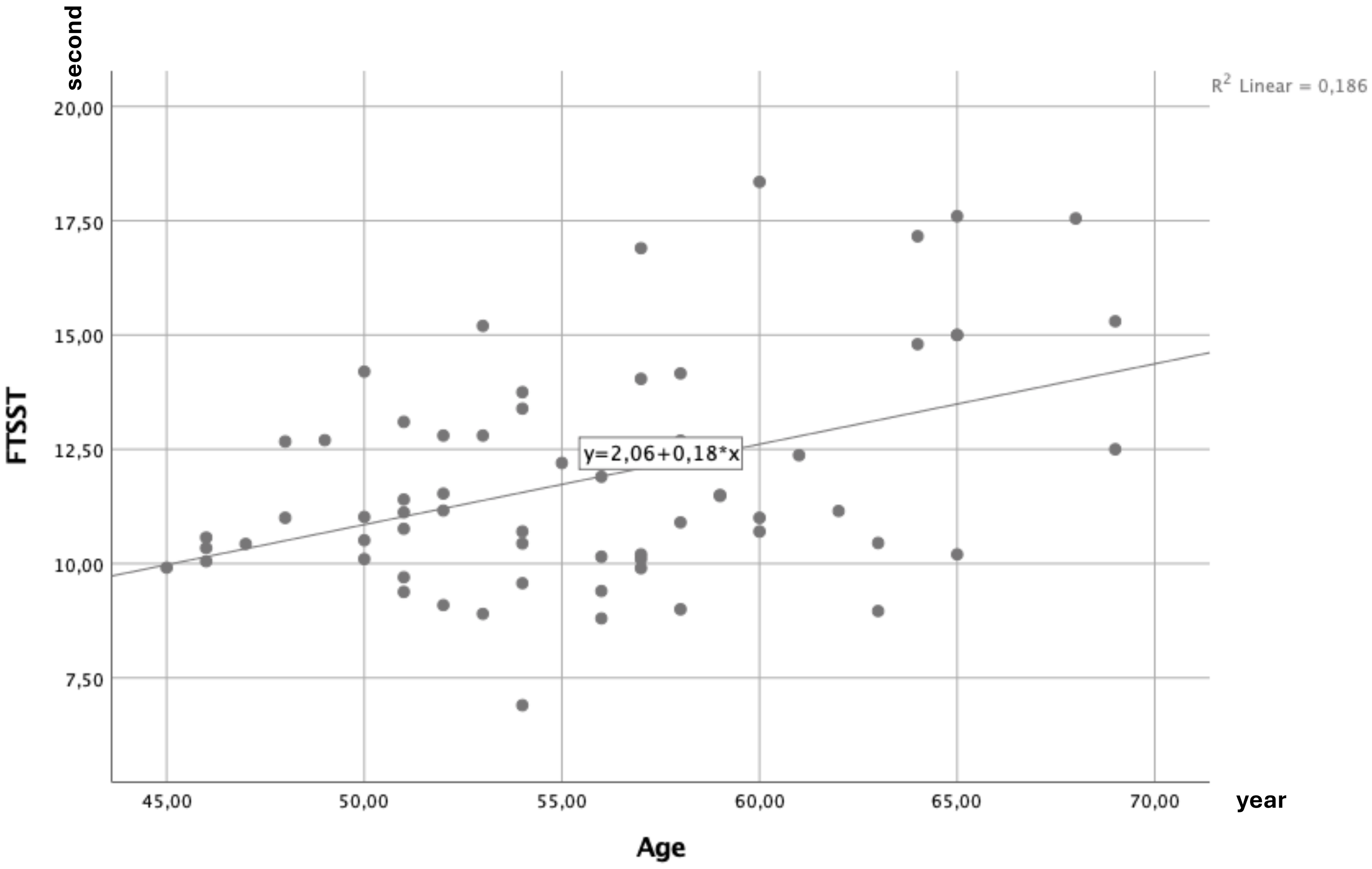

Figure S4: Correlation between FTSST and age (r=0.432, p <0.001)

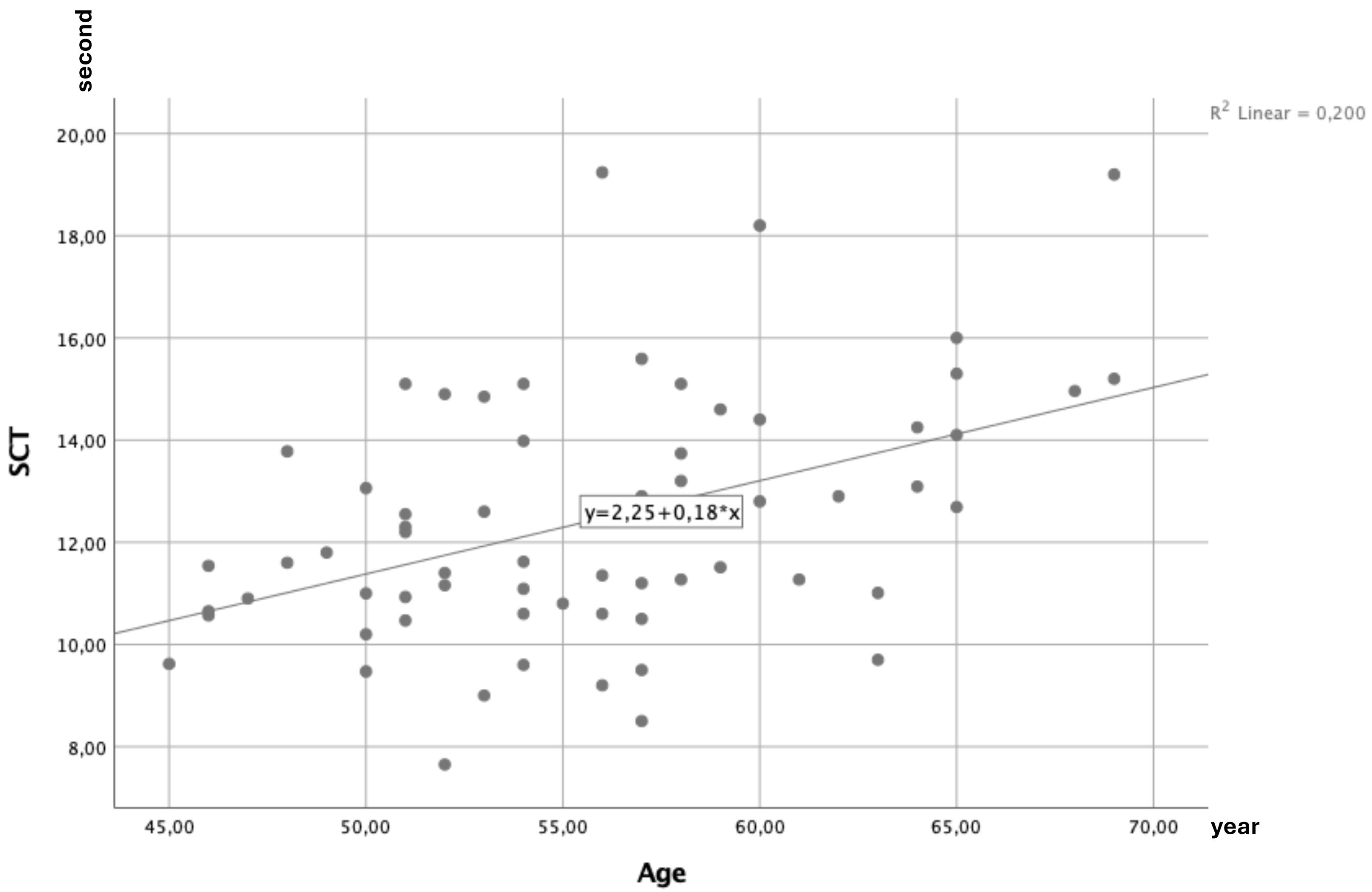

Figure S5: Correlation between age and the Stair Climb Test (SCT) (r=0.447, p <0.001)

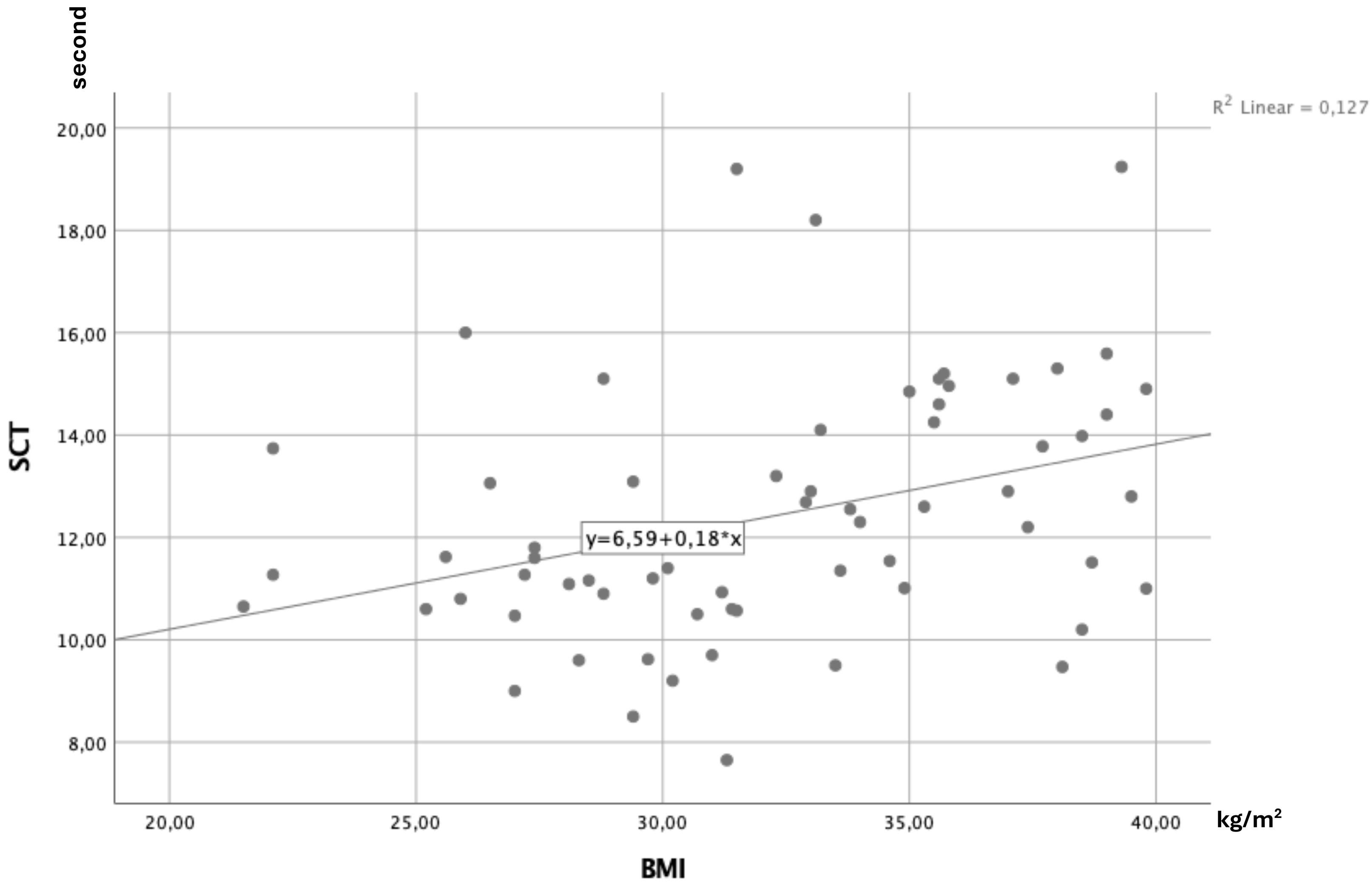

Figure S6: Correlation between BMI and SCT (r=0.356, p=0.004)

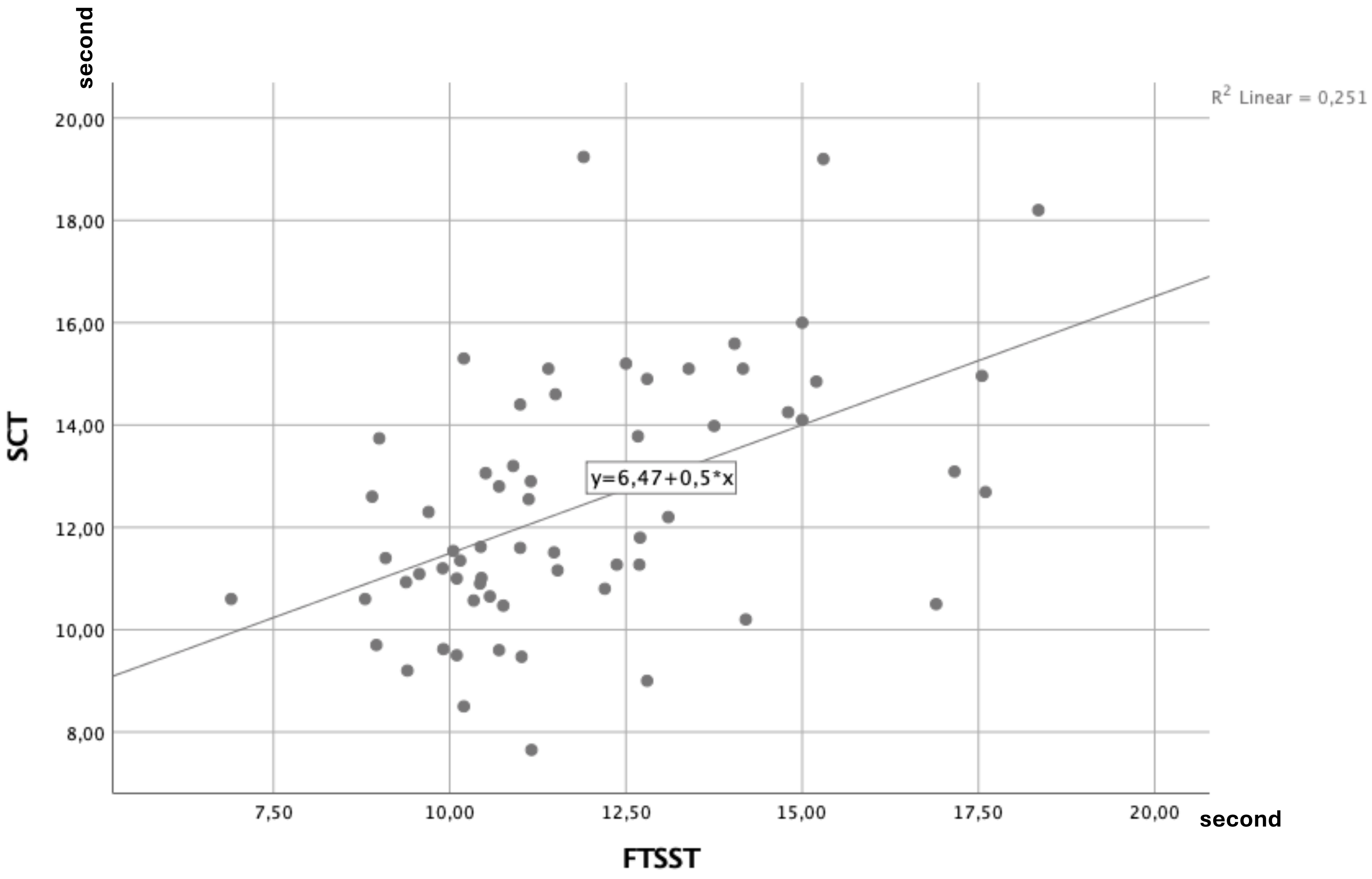

Figure S7: Correlation between FTSST and SCT (r=0.501, p=0.001)

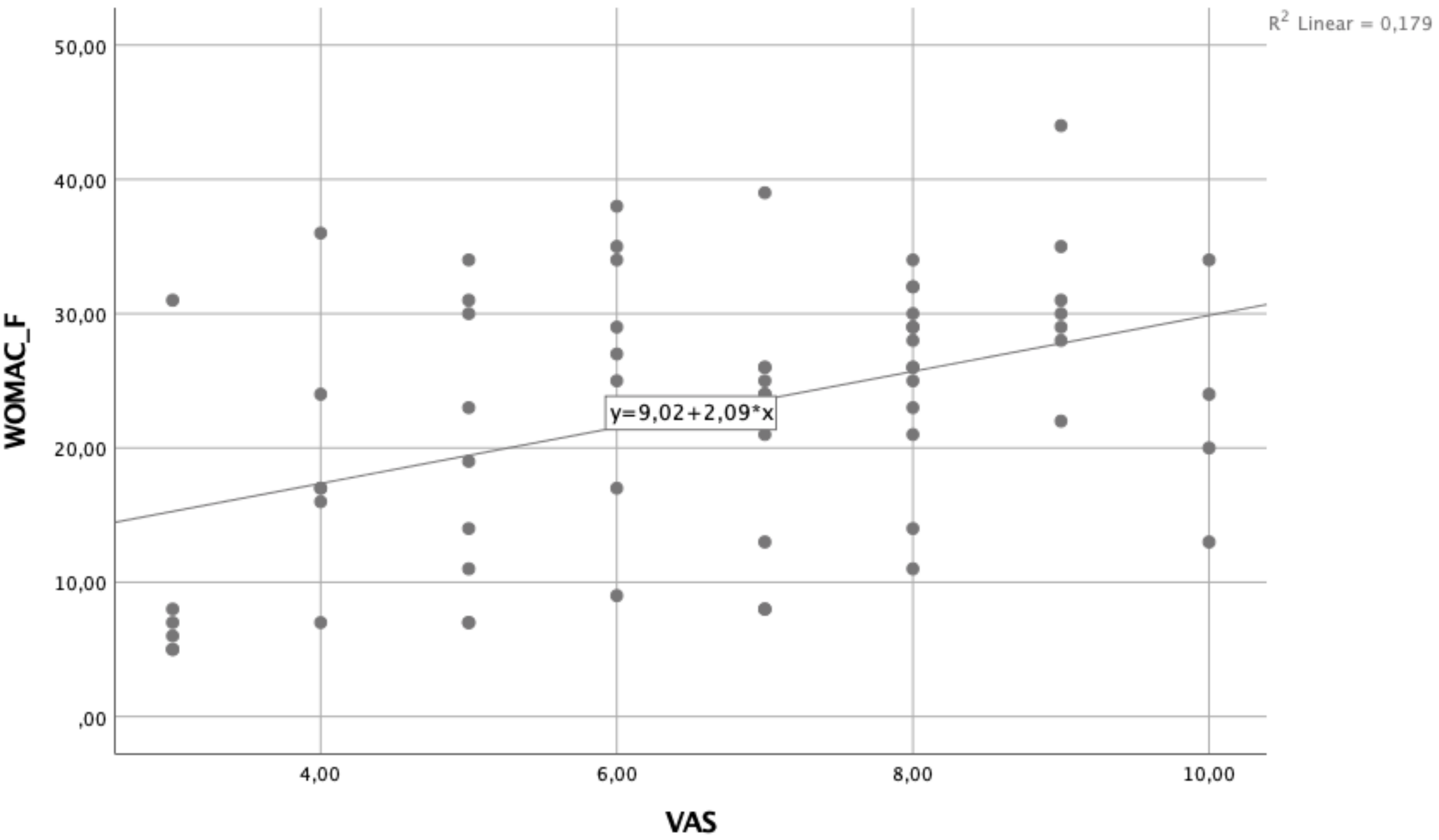

Figure S8: Correlation between VAS and WOMAC-F (r=0.424, p <0.001)

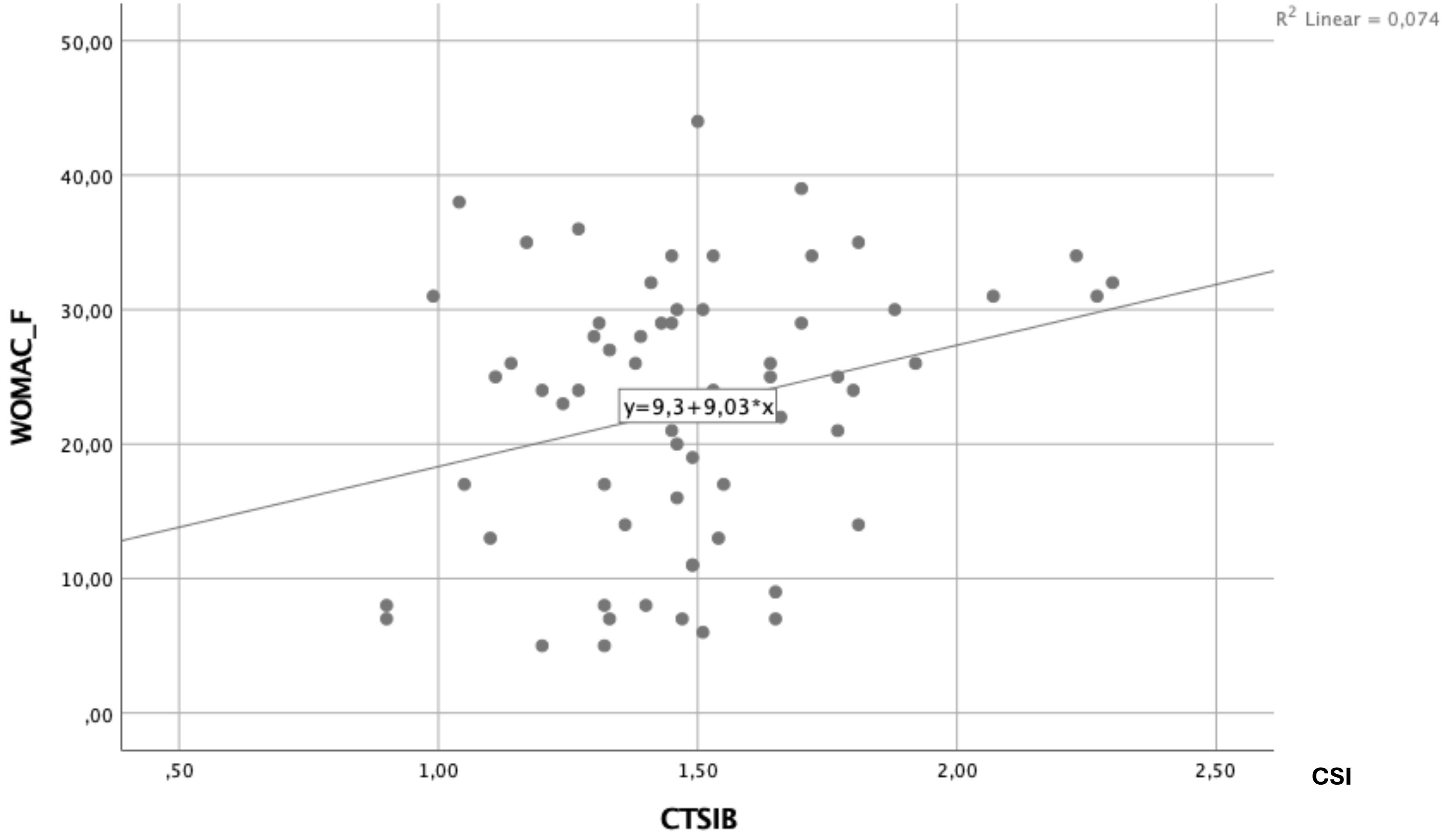

Figure S9: Correlation between CTSIB and WOMAC-F (r=0.271, p=0.030)
